# Bifunctional TRPV1 Targeted Magnetothermal Switch to Attenuate Osteoarthritis Progression

**DOI:** 10.34133/research.0316

**Published:** 2024-02-16

**Authors:** Zhongyang Lv, Peng Wang, Weitong Li, Ya Xie, Wei Sun, Xiaoyu Jin, Ruiyang Jiang, Yuxiang Fei, Yuan Liu, Tianshu Shi, Hu Guo, Ziying Sun, Jintao Lin, Xucai Wang, Guihua Tan, Yizhang Wu, Nirong Bao, Dongquan Shi

**Affiliations:** ^1^ Division of Sports Medicine and Adult Reconstructive Surgery, Department of Orthopedic Surgery, Nanjing Drum Tower Hospital, Affiliated Hospital of Medical School, Nanjing University, Nanjing 210008, China.; ^2^ Department of Orthopedics, Nanjing Jinling Hospital, Affiliated Hospital of Medical School, Nanjing University, Nanjing 210002, China.; ^3^ Division of Sports Medicine and Adult Reconstructive Surgery, Department of Orthopedic Surgery, Nanjing Drum Tower Hospital Clinical College of Nanjing University of Chinese Medicine, 321 Zhongshan Road, Nanjing 210008, Jiangsu, China.; ^4^ Department of orthopedic, The Jiangyin Clinical College of Xuzhou Medical University, Jiangyin 214400, China.; ^5^ Division of Sports Medicine and Adult Reconstructive Surgery, Department of Orthopedic Surgery, Nanjing Drum Tower Hospital Clinical College of Xuzhou Medical University, 321 Zhongshan Road, Nanjing 210008, Jiangsu, China.; ^6^ Co-Innovation Center for Efficient Processing and Utilization of Forest Resources, College of Chemical Engineering, Nanjing Forestry University, Nanjing, 210037, China.; ^7^ Department of Applied Physical Sciences, The University of North Carolina at Chapel Hill, Chapel Hill, NC, USA.

## Abstract

Transient receptor potential vanilloid family member 1 (TRPV1) has been revealed as a therapeutic target of osteoarthritis (OA), the most common deteriorating whole joint disease, by impeding macrophagic inflammation and chondrocytes ferroptosis. However, the clinical application for capsaicin as the TRPV1 agonist is largely limited by its chronic toxicity. To address this issue, we developed a bifunctional controllable magnetothermal switch targeting TRPV1 for the alleviation of OA progression by coupling of magnetic nanoparticles (MNPs) to TRPV1 monoclonal antibodies (MNPs-TRPV1). Under the alternating magnetic field (AMF) stimulation, MNPs-TRPV1 locally dissipated heat, which was sufficient to trigger the opening and activation of TRPV1, and effectively impeded macrophagic inflammation and chondrocyte ferroptosis. This magnetothermal modulation of TRPV1 simultaneously attenuated synovitis and cartilage degeneration in mice incurred by destabilization of medial meniscus surgery, indicating the delayed OA progression. Furthermore, MNPs-TRPV1 with AMF exposure remarkably reduced knee pain sensitivity, alleviated the crippled gait, and improved spontaneous ambulatory activity performance in the mice OA model. Overall, this work provides a potential pathogenesis-based precise OA therapy with temporally and spatially magnetothermal modulation of TRPV1 in a controllable manner.

## Introduction

Osteoarthritis (OA) is the most common deteriorating whole joint disease, with symptoms of chronic joint pain and disability [1]. The prevalence of OA is steadily increasing over the past 30 years, affecting approximately 5% population globally and leading to a robust increase of socioeconomic burden [2,3]. Therefore, due to no disease-modifying strategy currently available [4], there is a tremendous need to exploit therapeutic approaches targeting OA pathogenesis to alleviate disease progression.

OA is a degenerative joint disease involves pathological changes of whole joint compartments: cartilage, synovium, and subchondral bone [5]. Recent advances in OA basic research reported that cartilage degeneration caused by ferroptosis of chondrocytes [6] and synovitis triggered by macrophagic inflammation [7] were responsible for the deterioration of OA. We have previously corroborated that the transient receptor potential vanilloid family member 1 (TRPV1), a heat-sensitive channel, was highly colocalized to inflammatory macrophage markers in synovium, and pharmacological activation of TRPV1 by its specific agonist, capsaicin, remarkably impeded macrophagic inflammation and subsequent synovitis [8]. Moreover, TRPV1 was expressed in chondrocytes, and its activation evidently protected chondrocytes from ferroptosis and alleviated articular cartilage degradation by upregulating glutathione peroxidase 4 (GPX4), a typical ferroptosis suppressor [9]. These bifunctional role of TRPV1 synergistically attenuated OA progression. Although our previous works provided promising therapeutic target for OA, capsaicin injection as TRPV1 activator in clinical applications might be restricted by its toxic side effects, for instance, persistent desensitization and skin irritation [10]. In addition, highly diffusible capsaicin in joint cavity is incapable to stimulate TRPV1 in a temporally and spatially controlled manner [11]. Thus, accurately manipulating TRPV1 in living system remains a challenge, and the resolution of this issue may provide innovative approaches for OA treatment.

Nanoparticles at high temporal and spatial resolutions have been developed to precisely regulate cell signaling pathways [12,13]. The adoption of nanomaterials usually relies on external stimulus including electromagnetic induction [14] and optical [11] or acoustic signals [15], which are largely hindered by tissue absorption and scattering. Whereas, in contrast, low-radiofrequency (100 kHz to 1 MHz) alternating magnetic field (AMF) have become a powerful and versatile tool for the precise modulation of cell signaling pathways due to their deep penetration into body without attenuation or causing adverse physiological effects [16–18]. Considering the thermal sensitivity of TRPV1, which opens when the surrounding temperature exceeds 42 °C, researchers have been attempting to achieve controllable magnetothermal modulation of TRPV1 using magnetic nanoparticles (MNPs) since 2012 [16]. In the condition of exogenous TRPV1 expression, the neuromodulation of deep brain was achieved by MNPs dissipated heat under the exposure of AMF [19,20]. Furthermore, magnetothermal stimulation approach regulated adrenal hormones secretion in a transgene-free mode because of endogenously expressed TRPV1 in adrenal cortex and medulla [21]. Unlike in brain and adrenal tissues, which are substantial structures, the untargeted MNPs may not suitable for OA treatment due to the cavity structure of joint. Intraarticular injected MNPs are difficult to accumulate in the lesions within OA joint, especially the aforementioned inflammatory macrophages in the synovium and ferroptotic chondrocytes in the articular cartilage. Moreover, due to the high heterogeneity of articular cells, untargeted MNPs may exhibit unpredictable toxic side effects on disease-free cells in the AMF field. Hence, endowing MNPs to intelligently target TRPV1^+^ inflammatory macrophages and ferroptotic chondrocytes may provide a therapeutic avenue for pathogenesis-based OA therapy.

**Fig. 1.**
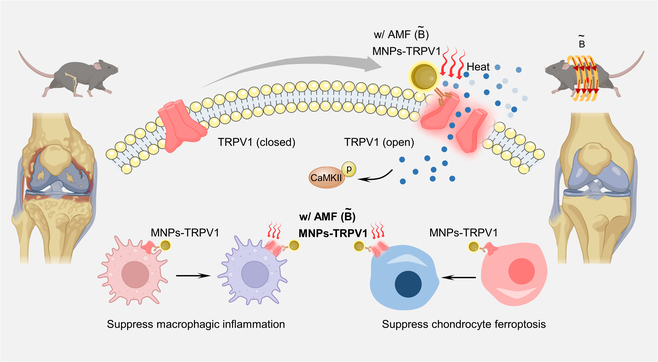
Illustration of bifunctional MNPs-TRPV1 magnetothermal switch for the activation of TRPV1 to attenuate osteoarthritis progression.

Herein, to solve the above problems, we developed a TRPV1 monoclonal antibody coupled MNPs (MNPs-TRPV1) as a magnetothermal switch for the controllable activation of TRPV1 to alleviate OA progression (Fig. 1). The TRPV1 monoclonal antibody enabled specific binding of MNPs-TRPV1 to TRPV1 expressed on the plasma membrane of macrophages and chondrocytes. Upon exposure to AMF fields, the local heat dissipated by MNP-TRPV1 was sufficient to open TRPV1, which effectively suppressed macrophagic inflammation and chondrocytes ferroptosis and ultimately attenuated OA progression. This work may provide a novel pathogenesis-based OA therapy, while offering a precise and generalizable approach for the controllable modulation of TRPV1 activation.

## Results and Discussion

### Preparation and characterization of MNPs-TRPV1

**Fig. 2.**
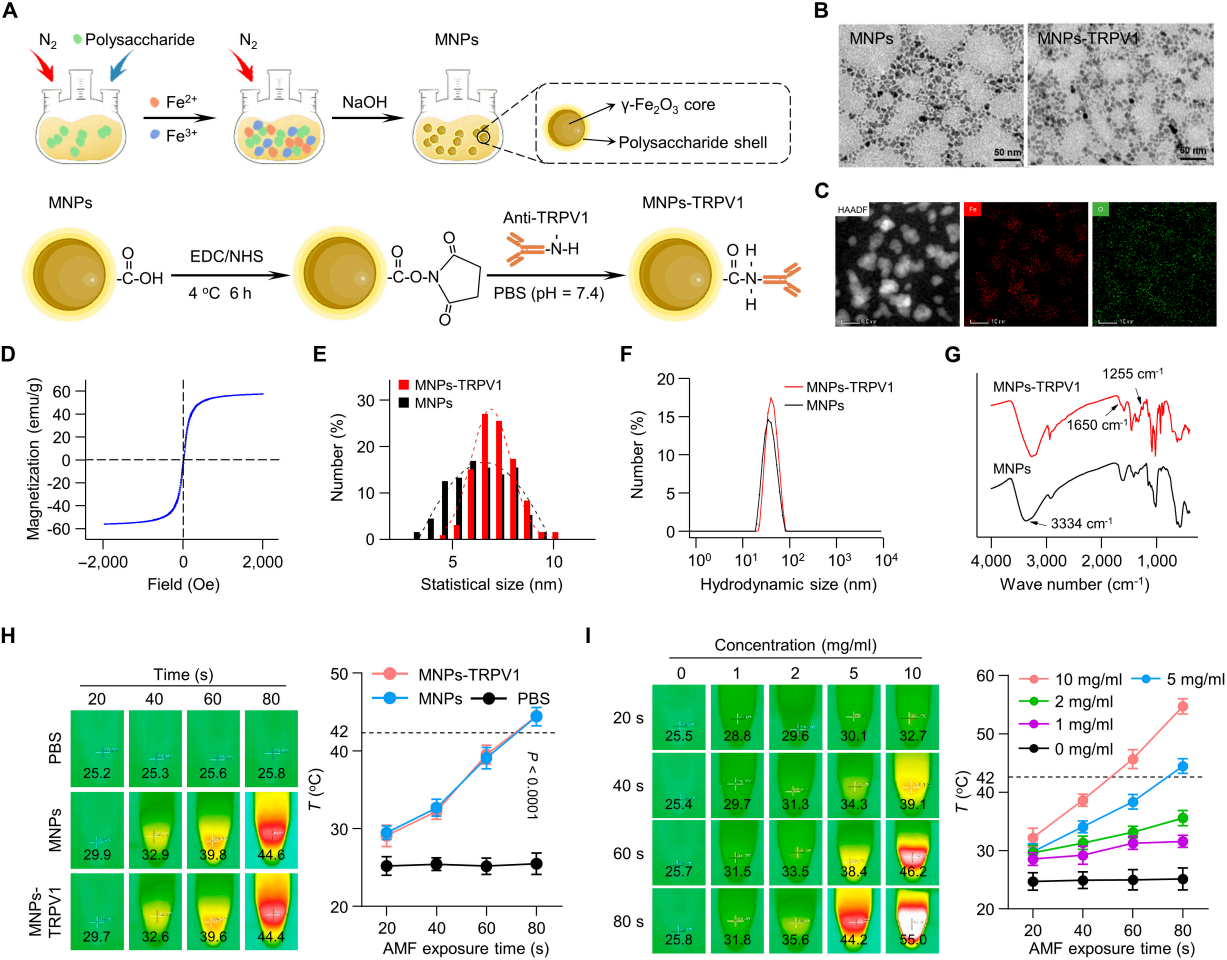
Preparation and characterization of MNPs-TRPV1. (A) Schematic diagram of the synthesis of MNPs-TRPV1. (B) TEM images of MNPs and MNPs-TRPV1. (C and D) Elemental mapping (C) and hysteresis loop (D) of MNPs. (E to G) Statistical size (E), hydrodynamic size (F), and FT-IR spectra (G) of MNPs and MNPs-TRPV1. (H) Heating curves of the PBS, MNPs, and MNPs-TRPV1 upon various AMF exposure time. (I) Heating curves of various concentrations of MNPs-TRPV1 upon various AMF exposure time. The dotted line represents activation threshold (42 °C) of TRPV1.

MNPs were firstly prepared via classic chemical coprecipitation method using polysaccharide as a stabilizer and subsequently TRPV1 monoclonal antibodies were conjugated to their surface through a condensation reaction between the amino groups of the TRPV1 monoclonal antibody and the carboxyl groups of the MNPs, resulting in the formation of MNPs-TRPV1 (Fig. 2A). Transmission electron microscopy (TEM) images revealed that both MNPs and MNPs-TRPV1 were of uniform dispersity and the conjugation of TRPV1 antibody minimally impact their morphology (Fig. 2B). Element mapping images of MNPs demonstrated that Fe and O elements were uniformly distributed in the microsphere (Fig. 2C). Moreover, hysteresis loop of MNPs was detected using vibrating sample magnetometer and the results were demonstrated in Fig. 2D, in which the as-prepared MNPs were super-paramagnetic with the saturation magnetization up to 56 emu/g. Their statistical size according to the TEM images (Fig. 2E) demonstrated that there was no marked difference among MNPs (6.76 ± 1.42 nm) and MNPs-TRPV1 (6.93 ± 0.98 nm). Data obtained from dynamic light scattering measurements (Fig. 2F) demonstrated that the mean hydrodynamic size of MNPs-TRPV1 was 32.9 nm, which was slightly larger than that of MNPs (28.6 nm). This variation could be attributed to the conjugation of the TRPV1 monoclonal antibody. Furthermore, their chemical structures, as characterized by Fourier transform infrared analysis (Fig. 2G), revealed that the presence of stretching bands at 1,650 and 1,255 cm^−1^ were associated with the stretching of C=O-NH bonds, confirming the formation of amide bonds and successful conjugation of TRPV1 monoclonal antibody.

To evaluate the magnetothermal conversion efficiency, MNPs and MNPs-TRPV1 solutions (5 mg/ml) were exposed upon AMF for 80 s and the heating curves were recorded. As illustrated in Fig. 2H, the temperature could be approximately heated to 44 °C by MNPs-induced magnetothermal effects upon AMF. Similar trend could be observed for MNPs-TRPV1, indicating that the conjugation of anti-TRPV1 barely affect the magneto-thermal conversion performance of MNPs. Notably, such ability to rise surrounding temperature above 42 °C within a short period of time endows MNPs-TRPV1 with the ability to activate TRPV1. Furthermore, MNPs-TRPV1 demonstrated a concentration-dependent increase in magnetothermal conversion efficiency, with the heating temperature reaching 31.8 °C at 1 mg/ml, 35.6 °C at 2 mg/ml, 44.2 °C at 5 mg/ml, and 55.0 °C at 10 mg/ml for 80 s of AMF exposure (10 A) (Fig. 2I). Moreover, the magnetothermal conversion efficiency of MNPs-TRPV1 could also be sensitively regulated by altering the intensity of AMF (Fig. S1). These results indicate that precise control of the desired heating temperature can be achieved by adjusting solution concentration, AMF exposure time, and AMF intensity, holding significant promise for the controlled magnetothermal modulation of MNPs-TRPV1.

### Magnetothermal suppression of macrophagic inflammation and chondrocyte ferroptosis by MNPs-TRPV1

**Fig. 3.**
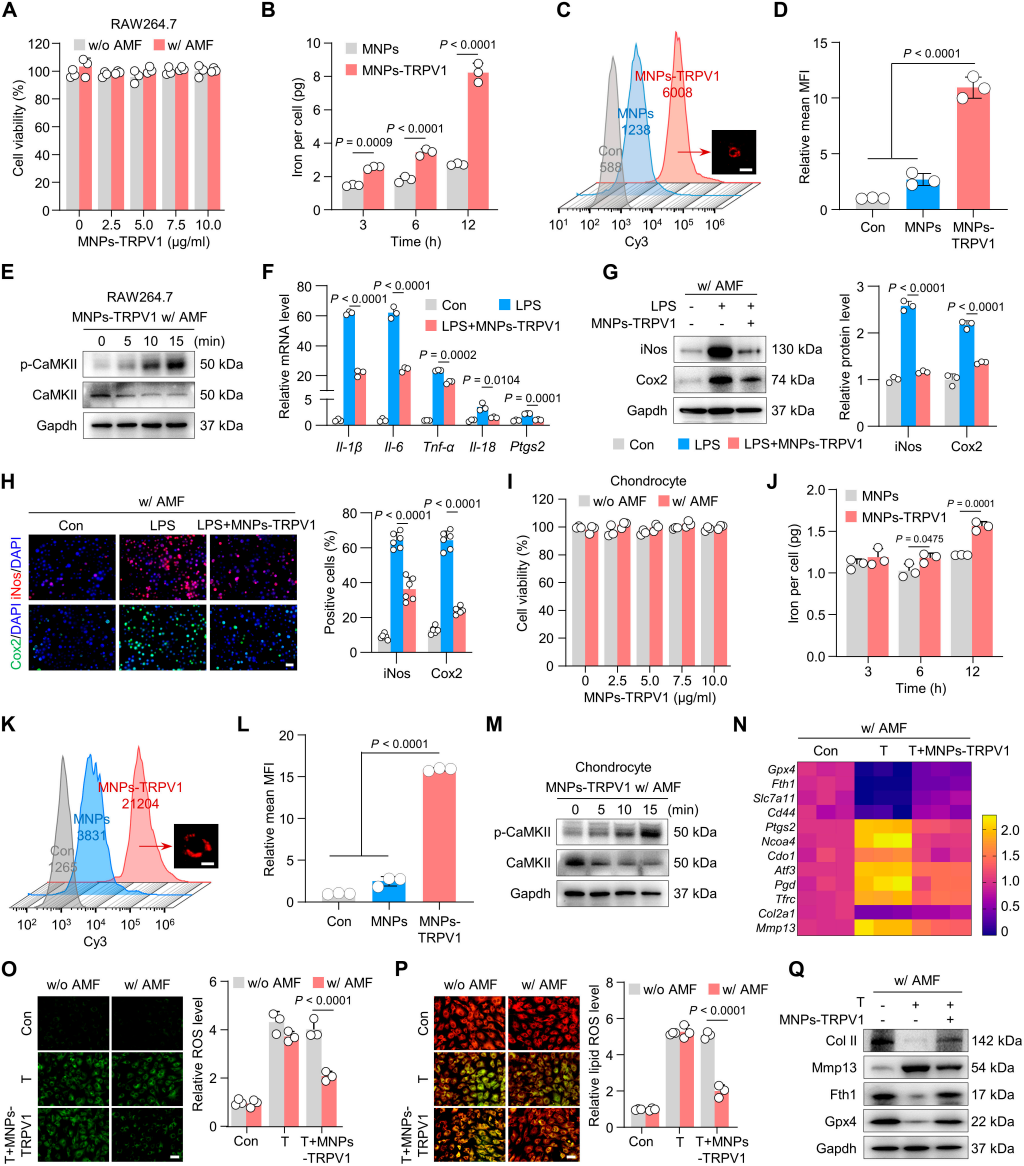
Magnetothermal suppression of macrophagic inflammation and chondrocyte ferroptosis by MNPs-TRPV1. (A) Cell viability of RAW264.7 macrophages treated with different concentrations of MNPs-TRPV1 with (w/) or without (w/o) AMF stimulation. (B) The inductively coupled plasma mass spectrometry (ICP-MS) detection of iron content per RAW264.7 cell after incubated with 5 mg/ml MNPs or MNPs-TRPV1 for 3, 6, and 12 h. (C and D) Flow cytometry analysis (C) and quantitative analysis (D) of sulfo-cyanine3 (Cy3) conjugated MNPs or MNPs-TRPV1 incubated RAW264.7 cells for 12 h. The representative image presented in (C) showed the combination of MNPs-TRPV1 to the plasma membrane of RAW264.7 cell. (E) Western blot analysis of the expression levels of CaMKII and p-CaMKII in RAW264.7 cells 0, 5, 10, and 15 min after the treatment of MNPs-TRPV1 with AMF stimulation. (F) qPCR analysis of inflammatory genes, including *Il-1β*, *Il-6*, *Tnf-α*, *Il-18*, and *Ptgs2*, in RAW264.7 cells treated with or without 50 ng ml^−1^ LPS in the presence or absence of MNP-TRPV1 pretreatment under AMF exposure. (G) Western blot analysis (left panel) and corresponding quantitative analysis (right panel) of the expression of inflammatory proteins, iNos and Cox2, expressed in RAW264.7 cells induced as indicated. (H) Immunofluorescence staining (left panel) and quantitative analysis (right panel) of iNos and Cox2 in RAW264.7 cell induced as indicated. (I) Cell viability of mouse primary chondrocytes treated with various concentrations of MNPs-TRPV1 with (w/) or without (w/o) AMF stimulation. (J) The ICP-MS detection of iron content per chondrocyte after incubated with 5 mg/ml MNPs or MNPs-TRPV1 for 3, 6, and 12 h. (K and L) Flow cytometry analysis (C) and quantification (D) of Cy3-conjugated MNPs or MNPs-TRPV1 incubated chondrocytes for 12 h. The representative image presented in (K) showed the combination of MNPs-TRPV1 to the plasma membrane of chondrocyte. (M) The proteins expression levels of CaMKII and p-CaMKII in chondrocytes treated as indicated at the time point of 0, 5, 10, and 15 min. (N) qPCR analysis of gene expression levels of ferroptosis suppressors (*Gpx4*, *Fth1*, *Slc7a11*, and *Cd44*), ferroptosis drivers (*Ptgs2*, *Ncoa4*, *Cdo1*, *Atf3*, *Pgd*, and *Tfrc*), cartilage anabolic marker (*Col2a1*), and cartilage catabolic marker (*Mmp13*) in chondrocytes treated as indicated. (O and P) Representative images (left panel) and quantification (right panel) of ROS (O) and lipid ROS (P) levels in mouse primary chondrocytes treated with TBHP (T) or T + MNPs-TRPV1 under the stimulation of AMF. (Q) Western blot analysis of expression levels of Col II, Mmp13, Fth1, and Gpx4 in chondrocytes treated as indicated. w/o, without; w/, with. Scale bars, 15 μm (C and K), 20 μm (H) and 50 μm (O and P). One-way (D, F, G, H, and L) or 2-way (A, B, I, J, O, and P) ANOVA with Tukey’s post hoc test. Data are shown as mean ± SD.

Given the endogenously expressed TRPV1 in inflammatory macrophages and ferroptotic chondrocytes [8,9], and the excellent magnetothermal effect exhibited by MNPs-TRPV1, we next sought to investigate whether MNPs-TRPV1 regulates macrophagic inflammation and chondrocyte ferroptosis under AMF exposure. The macrophage cell line, RAW264.7, remained no obvious viability decrease for 24-h incubation of MNPs-TRPV1 with or without AMF stimulation (Fig. 3A). To confirm the binding affinity of MNPs-TRPV1, we measured the iron abundance via inductively coupled plasma mass spectrometry (ICP-MS) and found that the calculated iron content per macrophage was significantly increased after 12-h incubation of MNPs-TRPV1 (8.22 ± 0.56 pg comparing to MNPs group 2.73 ± 0.06 pg, *P* < 0.0001) (Fig. 3B). Further flow cytometry analysis and fluorescence microscopic imaging observed remarkably increased density of sulfo-cyanine3 (Cy3)–conjugated MNPs-TRPV1 in the plasma membrane of RAW264.7 cells after 12 h of incubation when compared to Cy3-conjugated MNPs (Fig. 3C and D and Fig. S2A). These results indicated that MNPs-TRPV1 exhibited a specific binding with the TRPV1 channel expressed on the membrane of macrophages, therefore, may result in efficient local heating.

To further investigate whether MNPs-TRPV1-dissipated local heat is sufficient to open TRPV1 channels, we measured the expression levels of phosphorylated calcium/calmodulin-dependent protein kinase II (p-CaMKII), a classical downstream protein reflecting TRPV1 activation [22]. With the application of AMF irradiation, the expression level of p-CaMKII was robustly upregulated in a time-dependent manner and increased approximately up to 6.9-fold after 15 min of treatment (Fig. 3E and Fig. S3A), suggesting that the AMF-induced MNPs-TRPV1 heating sufficiently triggered the open of TRPV1. Then, we tested the anti-inflammation effect of MNPs-TRPV1 by using the lipopolysaccharide (LPS) induced macrophagic inflammation model [23]. The increase in mRNA levels of several inflammatory cytokines, such as interleukin-1β (*Il-1β*), *Il-6*, *Il-18*, tumor necrosis factor-α (*Tnf-α*), and prostaglandin-endoperoxide synthase 2 (*Ptgs2*), induced by LPS was significantly mitigated by AMF-irradiated MNPs-TRPV1 (Fig. 3F). Further, treatment with MNPs-TRPV1 under field application remarkably suppressed the expression levels of inducible nitric oxide synthase (iNos) and Cyclooxygenase 2 (Cox2) (Fig. 3G and H), which are typical markers used to identify inflammatory macrophages [24], as well as reversed the changes of anti-inflammatory markers (Fig. S4).

Further, we investigated the binding and anti-ferroptotic effect of MNPs-TRPV1 on mouse primary chondrocytes. As expected, MNPs-TRPV1 did not exhibit obvious toxic effects on primary chondrocytes (Fig. 3I), and could efficiently bind to the chondrocytic plasma membrane with relative mean fluorescence intensity (MFI) of 15.88 ± 0.18 compared to Cy3-conjugated MNPs (2.49 ± 0.54) and control (1.00 ± 0.07) group (Fig. 3J to L and Fig. S2B). The level of p-CaMKII was also increased in AMF stimulated MNPs-TRPV1 induced chondrocytes in a time-dependent manner (Fig. 3M and Fig. S3B), coincident with the increase in RAW264.7 cells. The role of MNPs-TRPV1 in chondrocyte ferroptosis was then tested in the tert-butyl hydroperoxide (TBHP)-induced chondrocyte ferroptosis model [9]. Using quantitative polymerase chain reaction (qPCR) analysis of ferroptosis markers, we found that the genes encoding ferroptotic drivers (*Ptgs2*, *Ncoa4*, *Cdo1*, *Atf3*, *Pgd*, and *Tfrc*) were prominently increased and ferroptotic suppressors (*Gpx4*, *Fth1*, *Scl7a11*, and *Cd44*) were largely decreased by the treatment of TBHP, which were robustly converted by MNPs-TRPV1 under the AMF field application (Fig. 3N). Moreover, MNPs-TRPV1 significantly suppressed the gene expression of matrix metallopeptidase 3 (*Mmp13*), suggestive of cartilage catabolism, and increased the expression of collagen 2a1 (*Col2a1*), indicative of cartilage anabolism (Fig. 3N). Since ferroptosis is essentially lipid peroxidation that occurs in oxidative stress environments [25], we next examined the intercellular reactive oxygen species (ROS) and lipid peroxidation levels, revealing that the TBHP-induced ROS and lipid peroxidation levels were vastly diminished by MNPs-TRPV1 under AMF condition (Fig. 3O and P). Moreover, protein expression levels of Col II, Gpx4, and Fth1 were markedly up-regulated by MNPs-TRPV1 treatment, whereas Mmp13 was evidently down-regulated (Fig. 3Q and Fig. S5). Taken together, these results indicate that magnetothermal control of TRPV1 by MNPs-TRPV1 suppressed macrophagic inflammation and chondrocyte ferroptosis in vitro.

### MNPs-TRPV1 alleviates macrophagic inflammation and synovitis under AMF application

**Fig. 4.**
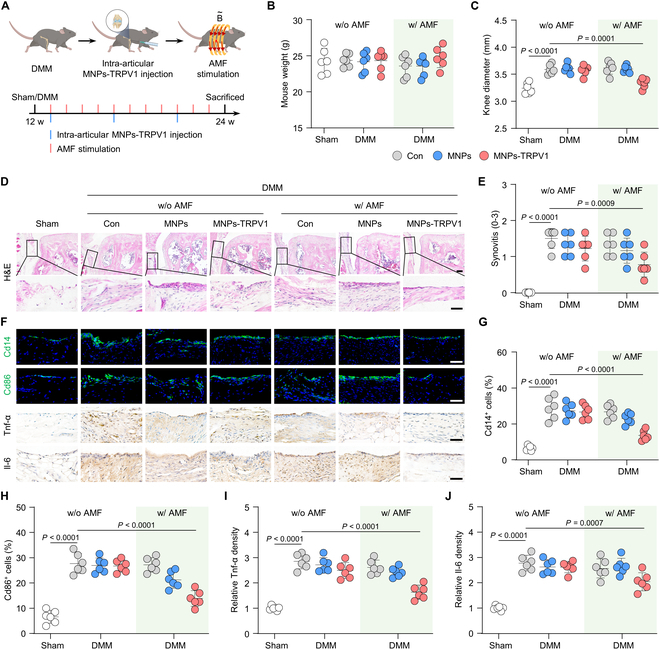
MNPs-TRPV1 suppresses macrophagic inflammation under AMF exposure in vivo. (A) Timeline for the intra-articular injection of MNPs-TRPV1 and subsequent AMF stimulation. (B and C) Mouse weight (B) and knee diameter (C) of mice at the endpoint of animal experiments. (D and E) Representative images (D) of H&E staining of mouse knee sections and the quantified synovitis scores (E). (F to J) Representative images (f) of immunofluorescence or immunohistochemical staining and quantification of Cd14^+^ (G) and Cd86^+^ (H) cells, as well as the relative Tnf-α (I) and Il-6 (J) density in the synovium. w/o, without; w/, with. Scale bars, 50 μm. One-way ANOVA with Tukey’s post hoc test. Data are shown as mean ± SD.

To gain a deeper understanding of the anti-macrophagic inflammation effect of MNPs-TRPV1 in vivo, we performed destabilization of medial meniscus (DMM) surgery to induce mouse OA model. The mice were randomly divided into 7 groups: (a) Sham; (b) DMM; (c) DMM with MNPs; (d) DMM with MNPs-TRPV1; (e) DMM under AMF stimulation; (f) DMM with MNPs under AMF stimulation; and (g) DMM with MNPs-TRPV1 under AMF stimulation. One week following surgery, a total volume of 8-μl MNPs or MNPs-TRPV1 (5 mg/ml) were intra-articular injected, with a frequency of once every 4 weeks, and subsequent AMF stimulation at a weekly frequency starting from the day after injection, allowing sufficient time for MNPs-TRPV1 binding to the TRPV1 on the plasma membrane (Fig. 4A). Examination of the mouse weight revealed indistinguishable changes among the 7 groups after 12 weeks of treatment (Fig. 4B). Whereas the mouse knee diameters in MNPs-TRPV1 with AMF group significantly reduced to 3.34 ± 0.09 mm when compared to DMM group with 3.59 ± 0.09 mm (*P* = 0.0001), there was no significant statistical difference among the other groups (Fig. 4C), suggesting the reduced joint swelling by MNPs-TRPV1 under AMF exposure.

We next focused on the evaluation of macrophagic inflammation and synovitis. Mice after DMM surgery for 12 weeks exhibited typical OA phenotype of synovitis with increased quantification of synovitis score up to 1.50 ± 0.28, as demonstrated by leucocyte infiltration adjacent to medial meniscus and thickened synovium membrane (Fig. 4D and E), as well as increased proportion of Cd14^+^ total macrophages (from 6.50 ± 1.30% to 28.75 ± 6.09%, *P* < 0.0001) and Cd86^+^ inflammatory macrophages (from 6.58 ± 2.56% to 27.67 ± 3.97%, *P* < 0.0001) (Fig. 4F to H). MNPs-TRPV1 under the AMF application exhibited an appreciable decrease of leucocyte infiltration and synovium thickness, which was quantified by reduced synovitis score of 0.78 ± 0.34 (*P* = 0.0009) (Fig. 4D and E). Moreover, the proportion of Cd14^+^ and Cd86^+^ macrophages were evidently decreased to 13.67 ± 2.84% and 13.83 ± 3.13%, respectively (Fig. 4F to H). Whereas, the percentage of Cd206^+^ macrophages, the well-recognized anti-inflammatory macrophages, was not significantly changed (Fig. S6). When compared to DMM group, no significant changes in synovitis score, the percentage of Cd14^+^ and Cd80^+^ macrophages were observed in the groups injected with MNPs with or without AMF stimulation and MNPs-TRPV1 with the absence of AMF (Fig. 4D to H). Further immunohistochemistry staining revealed substantially decreased density of Tnf-α and Il-6, 2 major players of OA inflammation that could be secreted by Cd86^+^ inflammatory macrophages [7], in the synovium of MNPs-TRPV1 with AMF group, indicating the largely suppressed inflammation (Fig. 4F, I, and J). Notably, considering that fibroblast-like synoviocytes residing in the synovium exhibited inflammatory phenotypes during OA progression [26,27], the decreased Tnf-α and Il-6 expression in subintimal layer of synovium, where synoviocytes distributed, indicated that MNPs-TRPV1 with AMF stimulation might suppressed synoviocytes inflammation. Together, these results suggest that magnetothermal activation of TRPV1 by MNPs-TRPV1 under the exposure of AMF effectively suppresses macrophagic inflammation and synovitis in vivo.

### MNPs-TRPV1 protects chondrocytes from ferroptosis under AMF stimulation

**Fig. 5.**
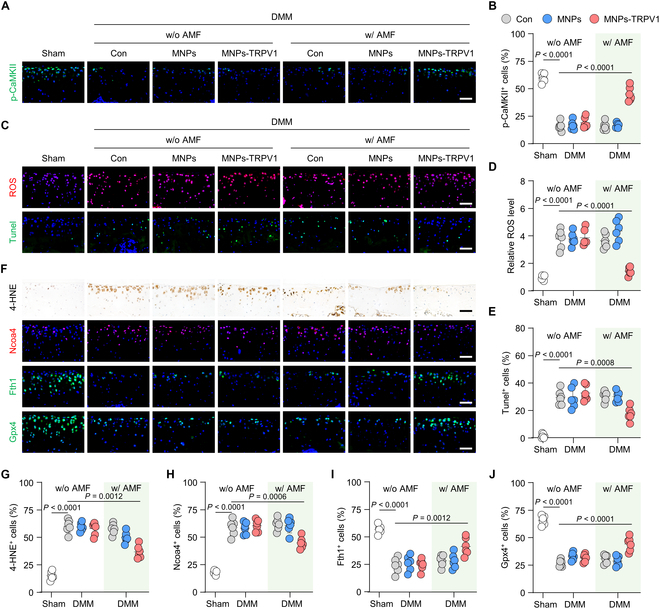
MNPs-TRPV1 protects chondrocytes from ferroptosis under AMF stimulation. (A and B) Representative images (A) and corresponding quantification (B) of the immunofluorescence staining of p-CaMKII in the mouse articular cartilage treated with MNPs or MNPs-TRPV1 with or without AMF exposure. (C to E) Measurement of ROS and Tunel^+^ chondrocytes (C), and their quantification (D and E) in the articular cartilage with the indicated treatment. (F to J) Representative images (F) of immunohistochemical or immunofluorescence staining and their quantification analysis of 4-HNE (G), Ncoa4 (H), Fth1 (I), and Gpx4 (J) in the mice cartilage treated with MNPs or MNPs-TRPV1 with or without AMF exposure. w/o, without; w/, with. Scale bars, 50 μm. One-way ANOVA with Tukey’s post hoc test. Data are shown as mean ± SD.

We then explored the role of MNPs-TRPV1 with AMF exposure in chondrocytes ferroptosis in vivo. To evaluate whether MNPs-TRPV1 tightly binds to articular cartilage, we performed ICP-MS detection and found that after 3 weeks injection, the iron concentration in the MNPs-TRPV1 group was evidently higher than in the MNPs group, indicating that MNPs-TRPV1 retained in the articular cartilage for at least 3 weeks (Fig. S7). The immunofluorescence staining exhibited impressively reduced expression of p-CaMKII in the cartilage of DMM group, suggestive of decreased TRPV1 activity, while MNPs-TRPV1 with AMF stimulation robustly increased the proportion of p-CaMKII^+^ chondrocytes (from 15.94 ± 3.87% in DMM group to 45.16 ± 6.46%, *P* < 0.0001), indicating the opening of TRPV1 triggered by MNPs-TRPV1 dissipated local heating (Fig. 5A and B). Due to the fact that ferroptosis is essentially ROS-stimulated lipid peroxidation [25], we detected the levels of ROS in articular cartilage and revealed that MNPs-TRPV1 with AMF reduced ROS levels to approximately 37.81% of those in the DMM group (Fig. 5C and D). Further, we directly presented dead chondrocytes by a terminal deoxynucleotidyl transferase dUTP nick-end labeling (Tunel) assay, which could visualize chondrocytes death-associated DNA fragmentation [28]. The percentage of Tunel^+^ chondrocytes significantly increased from 1.46 ± 0.62% in Sham group to 29.61 ± 5.31% in DMM group (*P* < 0.0001), while substantially decreased to 17.51 ± 4.86% (*P* = 0.0008) by MNPs-TRPV1 with AMF treatment (Fig. 5C and E), indicating the reduced chondrocyte death.

To further clarify the anti-ferroptosis effect of MNPs-TRPV1, we evaluated the levels of classical ferroptosis markers. Nuclear receptor coactivator 4 (Ncoa4)[29], a ferritinophagy mediator resulting in increased free iron, and 4-HNE, a prime lipid peroxidation produ ct [9], were both known as reflecting the occurrence of ferroptosis. Our data showed that the ratio of 4-HNE^+^ and Ncoa4^+^ chondrocytes were significantly decreased by MNPs-TRPV1 with AMF stimulation from 63.13 ± 6.05% to 39.74 ± 5.79% (*P* = 0.0012) and 58.76 ± 7.14% to 44.82 ± 5.13% (*P* = 0.0006) as compared to DMM group, respectively (Fig. 5F to H). The autophagic degradation of Ferritin heavy chain 1 (Fth1), a major intracellular protein that storage iron, leads to growing free iron accumulation and sensitizes cells to ferroptosis [30]. Comparing to DMM group, under the exposure of AMF, MNPs-TRPV1 substantially increased the proportion of Fth1^+^ chondrocytes from 24.03 ± 6.70% to 41.23 ± 7.97% (*P* = 0.0012) and Gpx4^+^ chondrocytes from 26.70 ± 3.12% to 44.20 ± 6.32% (*P* < 0.0001) (Fig. 5F, I, and J). These data indicate that MNPs-TRPV1 effectively protects chondrocytes from ferroptosis after stimulated by AMF fields.

### MNPs-TRPV1 under AMF stimulation alleviates OA progression

**Fig. 6.**
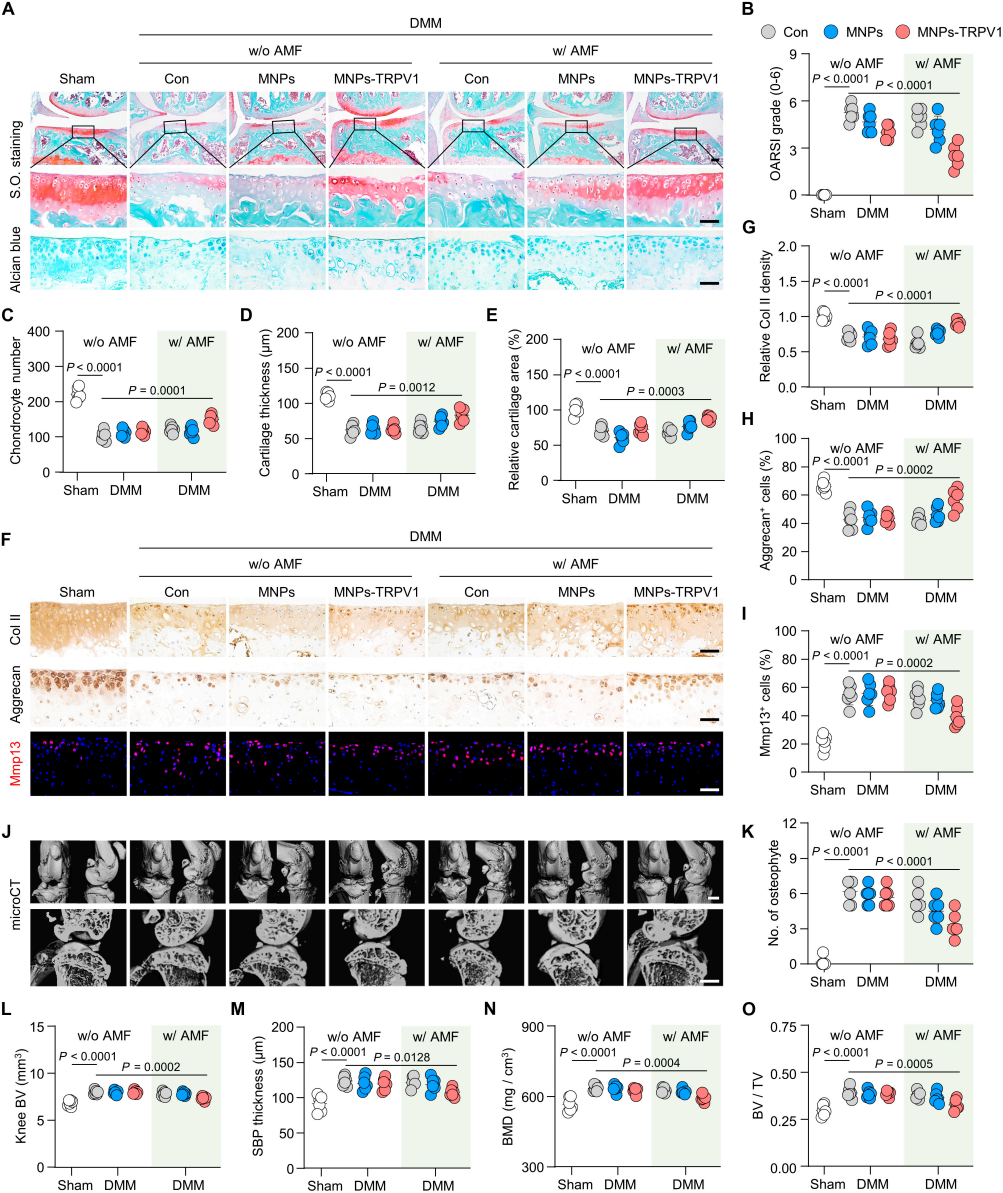
MNPs-TRPV1 under AMF stimulation alleviates OA progression. (A to E) Safranin-O/fast green (S.O.) and Alcian blue staining (A) and the corresponding quantification of Osteoarthritis Research Society International (OARSI) grade (B), chondrocyte number (C), cartilage thickness (D), and relative cartilage area (E) in the articular cartilage of mice treated by MNPs or MNPs-TRPV1 in the presence or absence of AMF exposure. (F to I) Immunohistochemical staining (F) and quantification of Col II (G) and Aggrecan (H), as well as immunofluorescence staining (F) and quantitative analysis (I) of Mmp13 in the cartilage of mice treated as indicated. (J) micro-CT analysis and 3D reconstructed images of mice knee joints, as well as the sagittal view of the medial joint compartment unveiling the changes to tibial and femoral surfaces and subchondral bone plate (SBP) thickness, respectively. (K to O) Quantitative analysis of the number (No.) of osteophyte (K), knee bone volume (BV) (L), SBP thickness (M), subchondral bone mineral density (BMD) (N), and the ratio of subchondral bone volume to tissue volume (BV/TV) (O) in the knee of mice treated as indicated. w/o, without; w/, with. Scale bars: 100 μm (upper panel of (A)), 50 μm (lower panel of (A) and (F)), and 1 mm (J). One-way ANOVA with Tukey’s post hoc test. Data are shown as mean ± SD.

Given the aforementioned anti-macrophagic inflammation and anti-chondrocytic ferroptosis role of MNPs-TRPV1, we then determined whether the AMF stimulated MNPs-TRPV1 attenuated OA progression ultimately. The mice underwent DMM surgery exhibited degenerative cartilage damage, including surface fibrillation, abnormal distribution of chondrocytes, and the loss of cartilage extracellular matrix, which were quantified by the substantially increased Osteoarthritis Research Society International (OARSI) grading, as well as decreased chondrocyte number, cartilage thickness, and relative cartilage area when compared with Sham group (Fig. 6A to E). Surprisingly, when compared to the DMM group, treatment with MNPs-TRPV1 under AMF exposure effectively attenuated the OARSI scores (from 5.25 ± 0.52 to 2.50 ± 0.71, *P* < 0.0001), improved the chondrocyte number (from 103.20 ± 13.86 to 148.50 ± 13.35, *P* = 0.0001), cartilage thickness (from 62.26 ± 7.29 μm to 83.22 ± 9.23 μm, *P* = 0.0012), and relative cartilage area (from 70.59 ± 6.54% to 88.39 ± 3.72%, *P* = 0.0003) (Fig. 6A to E). In addition, comparing with the DMM group, increased relative Col II density (from 0.72 ± 0.06 to 0.90 ± 0.04, *P* < 0.0001) and the percentage of Aggrecan^+^ chondrocytes (from 42.37 ± 6.51% to 56.73 ± 7.64%, *P* = 0.0002), suggesting cartilage anabolism, as well as decreased ratio of Mmp13^+^ chondrocytes (from 54.90 ± 7.28% to 39.23 ± 7.07%, *P* = 0.0002), indicating cartilage catabolism, were observed in MNPs-TRPV1 with AMF-treated mice (Fig. 6F to I). Whereas, treatment with AMF alone, MNPs with or without AMF application, and MNPs-TRPV1 in the absence of AMF did not noticeably alter the DMM-surgery induced cartilage degeneration and the expression of cartilage metabolic markers (Fig. 6A to I).

Furthermore, micro-computed tomography (micro-CT) analysis and subsequent 3-dimensional (3D) reconstructions of mice knee joints demonstrated abnormal bone remodeling in the DMM group, including narrowed joint space, uneven bone surface of the femur and tibia, as well as increased joint mineralization at the medial side, which was quantified by increased osteophyte number and knee bone volume (BV) when compared to the control group (Fig. 6J to L). These pathological alterations were significantly ameliorated by the treatment of MNPs-TRPV1 with AMF stimulation (Fig. 6J to L). Moreover, when focusing on the analysis of subchondral region, the DMM surgery induced increase of subchondral bone plate (SBP) thickness, bone mineral density (BMD), the ratio of subchondral bone volume to tissue volume (BV/TV), the number of trabecular (Tb.N), and the thickness of trabecular (Tb.Th) were significantly attenuated by the AMF-stimulated MNPs-TRPV1 (Fig. 6M to O and Fig. S8). Together, these results indicate that the intra-articular injected MNPs-TRPV1 under the AMF exposure could effectively attenuate OA progression.

### AMF-stimulated MNPs-TRPV1 alleviates knee pain and improves motor performance in mice

**Fig. 7.**
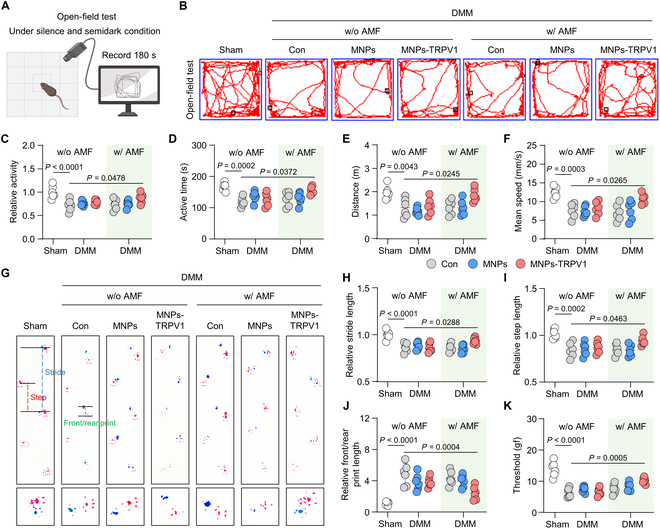
MNPs-TRPV1 improves behavior performance of mice. (A) Schematic illustration of the open field test. (B to F) Representative track plots of mice spontaneous activity in open field tests (B) and quantitative analysis of relative mice activity (C), active time during the 180 s experimental period (D), move distance (E), and mean speed (F). (G) Gait analysis of mice treated as indicated. Blue dot line, stride length; red dot line, step length; green dot line, front/rear print. Red print, fore paws; blue print, hind paws. (H and I) Quantification of mice foot prints including relative stride length (H), relative step length (I), and relative front/rear print length (J). (K) Mechanical sensitivity of mice was measured by von Frey tests. w/o, without; w/, with. One-way ANOVA with Tukey’s post hoc test. Data are shown as mean ± SD.

To investigate the functional improvement of mice knee by MNPs-TRPV1 with AMF, we performed a series of behavior tests. In silence and semidark condition, open field test records mouse movement trajectory and offers an objective read-out to quickly assess the general locomotor activity (Fig. 7A) [20]. Within the 180 s of testing time, we observed largely weakened motor performance of the DMM group when compared to the Sham group, as quantified by decreased locomotor activity, reduced active time, shortened active distance, and slowed mean speed (Fig. 7B to F). Under the treatment of MNPs-TRPV1 with AMF exposure, comparing to the DMM group, the mice motor performance was significantly improved with increased locomotor activity (from 0.72 ± 0.12 to 0.87 ± 0.10, *P* = 0.0478), prolonged active time (from 118.90 ± 15.11 s to 147.60 ± 12.20 s, *P* = 0.0372), extended active distance (from 1.31 ± 0.39 m to 1.84 ± 0.28 m, *P* = 0.0245), and faster mean speed (from 7.58 ± 1.88 mm/s to 10.72 ± 1.31 mm/s, *P* = 0.0265) (Fig. 7B to F).

Next, we conducted gait analysis of mice claudication [31]. During this process, the fore paws were stained with red ink and hind paws were stained with blue ink to record the footprints, allowing the mice to walk freely from one side to the other side on a 70 cm × 20 cm white track. Our results showed that the footprints of the hind and front paws relatively overlapped in the Sham group, while those obviously separated in the DMM group (Fig. 7G), suggesting increased knee pain after DMM surgery. Surprisingly, MNPs-TRPV1 with AMF exposure treatment significantly improved mouse gait, which was corroborated by the quantification of stride length, step length, and front/rear print length, when compared to DMM group (Fig. 7G to J). In addition, the results of von Frey tests exhibited remarkably higher paw withdrawal response thresholds after MNPs-TRPV1 and AMF treatment than that in the DMM group, suggesting reduced pain sensitivity (Fig. 7K). Thus, these data suggest that MNPs-TRPV1 with AMF exposure effectively alleviates knee pain and improves motor performance in mice.

**Fig. 8.**
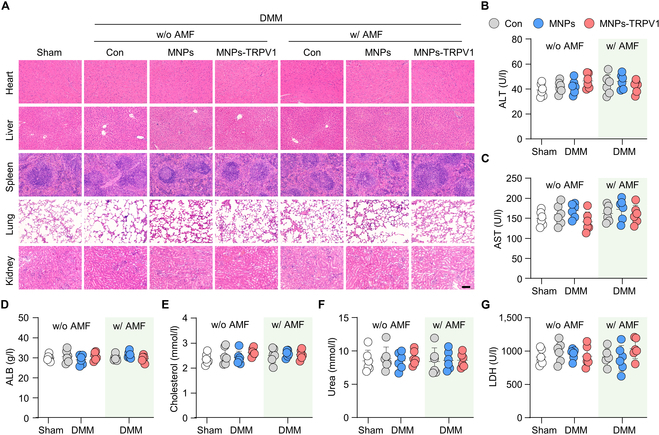
Safety measurement of MNPs-TRPV1. (A) H&E staining of the major organs (heart, liver, spleen, lung, and kidney) of mice treated as indicated revealed no obvious structural or cellular alterations. (B to G) The serum levels of alanine aminotransferase (ALT) (B), aspartate aminotransferase (AST) (C), albumin (ALB) (D), cholesterol (E), urea (F), and lactate dehydrogenase (LDH) (G) of mice treated with MNPs of MNPs-TRPV1 in the presence or absence of AMF stimulation. w/o, without; w/, with. Scale bars, 100 μm. One-way ANOVA with Tukey’s post hoc test. Data are shown as mean ± SD.

Moreover, in the postmortem analysis of mice heart, liver, spleen, lung, and kidney, no distinguishable structural or cellular changes were observed by hematoxylin and eosin (H&E) staining (Fig. 8A). The levels of alanine aminotransferase (ALT), aspartate aminotransferase (AST), albumin (ALB), cholesterol, urea, and lactate dehydrogenase (LDH) in the serum exhibited no significant alterations among the 7 groups (Fig. 8B to G), indicating the satisfactory biocompatibility and safety of the MNPs-TRPV1 with AMF stimulation.

## Conclusion

In summary, we have exploited an intelligent TRPV1 targeting bifunctional magnetothermal switch for the pathogenesis-based precise OA therapy. The coupling of TRPV1 monoclonal antibody conferred MNPs with the feature of precise integration to TRPV1 on the plasma membrane of macrophages and chondrocytes, without weakening the magnetothermal conversion. Under the stimulation of AMF, MNPs-TRPV1 dissipated local heat effectively triggered the opening of TRPV1, reflected by the time-dependent increase of p-CaMKII. As expected, AMF-stimulated MNPs-TRPV1 substantially abolished macrophagic inflammation and chondrocytes ferroptosis, resulting in a remarkable decrease of knee joint swelling, synovitis, cartilage extracellular matrix loss, and abnormal bone remodeling, as well as alleviated mice knee pain and improved motor performance. Thus, this study provides a novel TRPV1-targeting pathogenesis-based OA therapy in a controllable mode.

## Materials and Methods

### Preparation and characterization of MNPs-TRPV1

As previously described, MNPs were prepared by the classical chemical coprecipitation method, using polysaccharide as a stabilizer [32]. For the preparation of MNPs-TRPV1, we conjugated TRPV1 monoclonal antibody (#66983-1-Ig, Proteintech) to the surface of MNPs through a condensation reaction between the amino groups on the TRPV1 monoclonal antibody and the carboxyl groups on the MNPs. In brief, 575 μg of EDC and 346 μg of NHS were supplemented into a 1-ml solution of MNPs (0.5 mg/ml), then allowing them to react for 1 h under vigorous stirring at room temperature to activate the carboxylate groups on the surface of MNPs. Subsequently, 5 μg of TRPV1 monoclonal antibody was introduced to the solution and vigorously stirred at 4 °C for another 12 h. To eliminate free TRPV1 antibody, we conducted dialysis using membrane tubing with a molecular weight cutoff of 3000. The resulting MNPs-TRPV1 were sterilized via 0.22-μm membrane filters. For characterization, we conducted morphological observations using TEM (JEM-2100, Japan). Elemental mapping of MNPs was analyzed using high-angle annular dark-field scanning transmission electron microscopy (HAADF-STEM). The hydrodynamic size and Zeta (ζ) potential of MNPs-TRPV1 were evaluated through dynamic light scattering (Malvern Zetasizer Nano ZS90, UK). A Fourier transform infrared spectrometer (Nicolet is 50, Thermo) and a vibrating sample magnetometer (7407, Lakeshore) were employed to analyze their structure.

### Magnetothermal effect of MNPs-TRPV1

The AMF was generated by a voltage signal amplifier with frequency of *f* = 1.0 MHz (SPG-06-IV, Shuangping, Shenzhen, Country). The magnetothermal effect of MNPs-TRPV1 was evaluated by varied AMF exposure time (20 to 80 s), different concentrations (0 to 10 mg ml^−1^) of MNPs-TRPV1 that suspended in PBS, or irradiated by distinct AMF intensity (1 to 10 A). The temperature and thermal images were recorded by a thermal imager (MobIR).

### Cell culture

RAW264.7, a macrophage cell line, was purchased from the Cell Bank of Type Culture Collection of Chinese Academy of Science (Shanghai, China) and the mouse primary chondrocytes were collected from femoral heads of 3-d-old C57BL/6 mice as previously reported [9]. Cells were cultured in Dulbecco’s modified Eagle’s medium (Gibco, Carlsbad, CA) with the supplementation of 10% fetal bovine serum (Gibco) and 1% penicillin and streptomycin (Gibco) at 37 °C and 5% CO_2_ condition. To induce macrophagic inflammation, RAW264.7 cells were induced by 50 ng ml^−1^ lipopolysaccharide (LPS) (#L2630, Sigma-Aldrich, St. Louis, MO, USA). Chondrocytes were treated with 50 μM TBHP (#MKCH9944, Sigma, USA) to potentiate ferroptosis. After the incubation of 5 mg ml^−1^ MNPs or MNPs-TRPV1 for 12 h, LPS-induced RAW264.7 cells and TBHP-induced chondrocytes were subjected to AMF (10 A, *f* = 1.0 MHz) for 80 s to open TRPV1, and 12 h after treatment, the mRNA and total proteins were extracted for quantitative real-time polymerase chain reaction (qPCR) and Western blot analysis, respectively.

According to the manufacturer’s instructions, a Cell Counting Kit-8 (CCK8) assay (#CK04, Dojindo, Japan) was used to evaluate the cell viability. The combination of MNPs-TRPV1 to the plasma membrane was evaluated by the BD AccuriC6 Plus Flow CytoMeter (BD Biosciences, USA) and FlowJo software (version 10), as well as imaged with a fluorescence microscope (Zeiss, Germany). The evaluation of ROS and lipid ROS were performed in the TBHP + MNPs-TRPV1 induced chondrocytes pretreated with ROS probe 2',7'-dichlorodihydrofluorescein diacetate (10 μM) (#S0033S, Beyotime, China) and lipid ROS probe C^11^-BODIPY 581/591 (5 μM) (#GC40165, GLPBIO, USA), and the representative images were acquired by a fluorescence microscope (Zeiss, Germany) and quantified using ImageJ (version 1.8.0).

### Western blot analysis

Total proteins of RAW264.7 cells and primary chondrocytes were acquired by the RIPA lysis buffer (#R0010, Solarbio, China) with the supplement of 1 mM phosphatase inhibitor cocktail (#B15002, Bimake, USA) and 1 mM phenylmethanesulfonyl fluoride (#329-98-6, Solarbio). Western blot was executed as previously described [9]. The primary antibodies used in this work were: p-CaMKII (#ab124880, Abcam, USA), CaMKII (#ab134041, Abcam), iNos (#13120S, Cell Signaling Technology, USA), Cox2 (#12282S, Cell Signaling Technology), Col II (#BA0533, Boster, Wuhan, China), Mmp13 (#GB11247, Servicebio, China), Fth1 (#4393, Cell Signaling Technology), Gpx4 (#ab125066, Abcam), Cd206 (#18704-1-AP, Proteintech, China), Arg-1 (#16001-1-AP, Proteintech), and Gapdh (#5174, Cell Signaling Technology). After the incubation of horseradish peroxidase-conjugated goat anti-rabbit/mouse secondary antibodies (#BL003A or #BL001A, Biosharp), western blots were imaged by the ChemiDocXRS + Imaging System (Tanon, Shanghai, China). Quantification of proteins was performed by the ImageJ software (version 1.8.0). All primary and secondary antibodies used in this work were commercial.

### Quantitative real-time polymerase chain reaction

Cellular mRNAs from RAW264.7 cells and chondrocytes were isolated by RNA-quick Purification Kit (#RN001, ES Science, Shanghai, China) and HiScript-TS 5'/3' RACE Kit (RA101, Vazyme Biotech Co.,Ltd, China). The qPCR analysis was performed using the ChamQ Universal SYBR qPCR Master Mix (Q711, Vazyme Biotech Co.,Ltd) on a LightCycler 480 PCR System (Roche, Switzerland). The primer sequences are listed in Table S1.

### Animal experiments

All animal experiments were authorized and performed strictly in accordance with the Animal Care and Use Committee of Nanjing Drum Tower Hospital, The Affiliated Hospital of Nanjing University (2020AE01102). Male 12-week-old C57BL/6 mice were purchased from Model Animal Research Center of Nanjing University and kept in pathogen-free condition with free obtain of water and food. After Sham or DMM surgery on the right knee joint, mice were randomly divided into 7 groups (*n* = 6 per group): (a) Sham group; (b) DMM group; (c) DMM with MNPs injection group; (d) DMM with MNPs-TRPV1 injection group; (e) DMM with AMF exposure group; (f) DMM with MNPs injection and AMF exposure group; and (g) DMM with MNPs-TRPV1 injection and AMF exposure group. One week after surgery, intra-articular injection of 8-μl MNPs or MNPs-TRPV1 (5 mg ml^−1^) were performed, while the same volume of PBS was injected in the Sham and DMM group, with a frequency of once every 4 weeks. The AMF treatment (10 A, *f* = 1.0 MHz) started from the second day after injection and performed at a frequency of once a week. The number of mice in every group was calculated according to Charan and Kantharia proposed formula [33]. Our animal study strictly complied with the ARRIVE guideline.

The DMM surgery-constructed mice OA model was essentially conducted as previously reported [34]. Briefly, after anaesthetizing mice, we opened the articular cavity of right knee and sectioned the medial menisco-tibial ligament to destabilize the medial meniscus. We performed the same surgery on the right knee of the Sham group without ligament sectioning.

### Histological analysis

The mouse knee joints were harvested and decalcified by EDTA with a concentration of 10% (#1340, Biofroxx, Germany) solution, then were embedded in paraffin blocks. Following a previously reported procedure [35], mouse knee joints were prepared into continuous 5 μm coronal slides by a microtome (Thermo, Germany). After selecting every fifth of 2 slides, Safranin-O/fast green (S.O.) (#G1371, Solarbio) and H&E (#C0105S, Beyotime) staining were performed to assess the synovitis and cartilage degradation [36]. The synovitis score (0-3) and OARSI grading system (0-6) were used to assess inflammation and cartilage degeneration by 2 blinded observers [37]. The highest synovitis score and OARSI score were recorded and the averages of them were calculated. Moreover, H&E (#C0105S, Beyotime) staining of major organs (heart, liver, spleen, lung, and kidney) slides was performed to evaluate the biocompatibility of MNPs and MNPs-TRPV1 in vivo. The ROS levels and dead chondrocytes were evaluated by the tissue ROS detection assay (#BB-460522, Bestbio, China) and Fluorescein (FITC) Tunel Cell Apoptosis Detection Kit (#G1501, Servicebio) based on the manufacturer’s instructions, respectively.

### Immunofluorescence and immunohistochemical staining

After being deparaffinized, the mouse knee joint slides were blocked with 5% bovine serum albumin (BSA) for 1 h, and then treated with primary antibodies overnight (4 °C). The used primary antibodies for immunofluorescence and immunohistochemical staining were: Cd14 (#17000-1-AP, Proteintech), Cd86 (#13395-1-AP, Proteintech), Tnf-α (#60291-1-Ig, Proteintech), Il-6 (#21865-1-AP, Proteintech), p-CaMKII (#ab124880, Abcam), 4-HNE (#ab48506, Abcam), Ncoa4 (#66849, Cell Signaling Technology), Fth1 (#4393, Cell Signaling Technology), Gpx4 (#ab125066, Abcam), Col II (#BA0533, Boster), Aggrecan (#13880-1-AP, Proteintech), Mmp13 (#GB11247, Servicebio), and Cd206 (#18704-1-AP, Proteintech). The slides were then incubated with fluorescein isothiocyanate (FITC) or tetramethylrhodamine-5-(and 6)-isothiocyanate-conjugated secondary antibodies (Abcam) at room temperature (1 h) for immunofluorescence staining. A fluorescence microscope (Zeiss, Germany) was used to obtain the fluorescence images. For immunohistochemical staining, we used 3% (v/v) H_2_O_2_ to quench the activity of endogenous peroxidase. Horseradish peroxidase-conjugated anti-rabbit or anti-mouse immunoglobulin G (IgG) (Biosharp, Shanghai, China) were used as the second antibody. An ultra-sensitive DAB Kit (#1205250, Typing, Nanjing, China) was selected for the visualization of immunohistochemical staining. Notably, in process of immunofluorescence and immunohistochemical staining, we used nonimmune IgG as the negative control. Two observers under blind condition performed the quantification of immunofluorescence and immunohistochemical staining of synovium and articular cartilage.

### Micro-computed tomography analysis

As previously described [9], a VivaCT 80 scanner (Scanco Medical AG, Switzerland) (70-kVp source) was used for micro-CT scanning and subsequent 3D reconstruction was performed by Scanco Medical software (Scanco threshold: 220) to evaluate the bone mass of knee joint from different treated mice. From the 3D reconstructive images, we calculated the number of osteophytes. The subchondral bone plate thickness, knee BV, BMD, BV/TV, Tb.N, and Tb.Th were analyzed as previously reported [38].

### Behavior tests

One day before sacrificing the animals, the behavioral experiments were conducted. In the open field test, the square arena with dimension of 50 cm length × 50 cm width and surrounding walls of 25 cm in height was used. Under silence and semidark condition, the mouse movement trajectory was recorded by a tracking system (Zhenghua Technology, China). The open field trial duration lasted 180 s. In the gait analysis of claudication, mice were placed in a 70 cm × 20 cm white open gait arena, which allowed freely walk. By pre-dipping the mice hind paws with blue ink and fore paws with red ink, the footprints were recorded. Result measures were acquired by 2 independent observers under blinded condition. The pain sensitivity was evaluated by an electronic von Frey Anesthesiometer (IITC, Woodland Hills, USA) and the paw withdrawal mechanical threshold was recorded.

### Statistical analysis

GraphPad Prism (version 8.0) and SPSS (version 25.0) were used for statistical analysis. Quantitative results represent at least 3 independent experiments. In the process of analysis, no animals or samples were excluded. To estimate the homogeneity of variance and data normal distributions, Levene method and Shapiro–Wilk test were performed, respectively. One-way or 2-way analysis of variance (ANOVA) followed by Tukey’s post hoc tests were used for the comparation of mean values of more than 2 groups. Data in this work were showed as mean ± SD. Differences were regarded statistically significant when *P* < 0.05.

## Data Availability

Data available on request from authors.
