# Supplementary material for: Bifunctional TRPV1 Targeted Magnetothermal Switch to Attenuate Osteoarthritis Progression
Source: Research (Wash D C). 2024 Feb 16;7:0316. doi: 10.34133/research.0316 (PMC10871150; doi:10.34133/research.0316)
Supplement: Supplementary 1 — Figs. S1 to S8 Table S1 [file research.0316.f1.docx]

Supplementary Materials

**Supplementary figures**


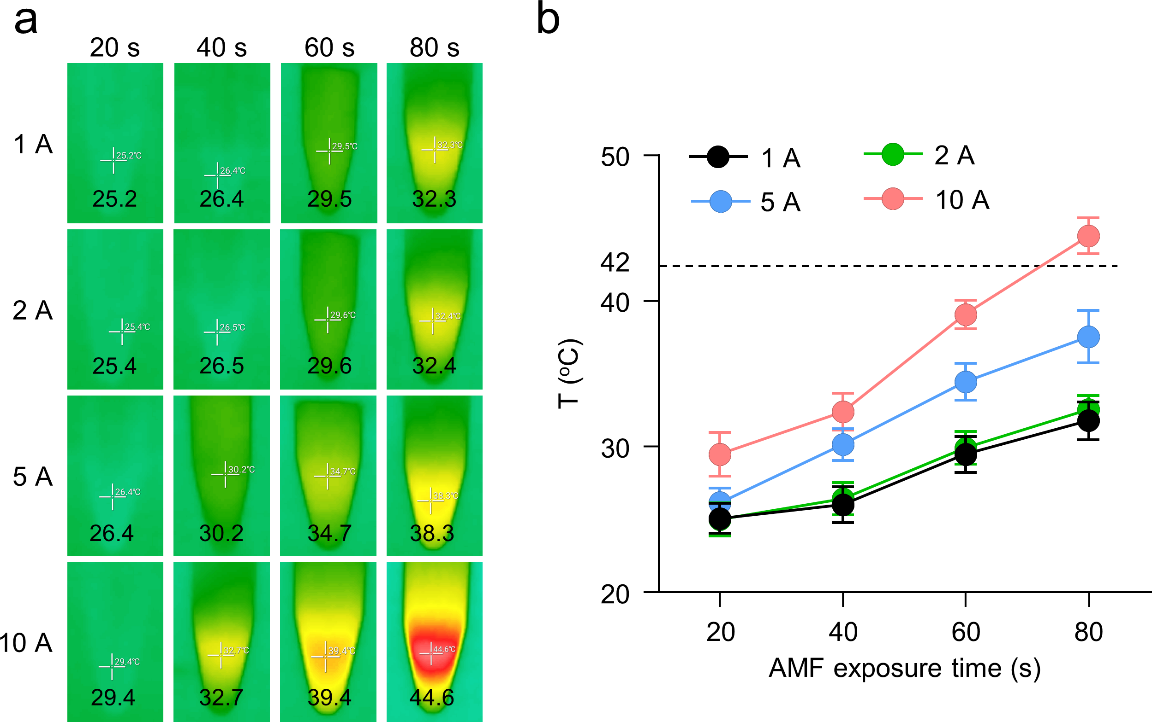


**Figure S1 The magnetothermal effect of MNPs-TRPV1. (a, b)** Representative images **(a)** obtained by a thermal camera and quantitative analysis **(b)** of temperature-rising curve of MNPs-TRPV1 irradiated by various density alternating magnetic field (AMF) (1 A, 2 A, 5 A, and 10 A) for different times (20 s, 40 s, 60 s, and 80 s). The dotted line represents activation threshold (42 ℃) of TRPV1. Data are shown as mean ± SD.


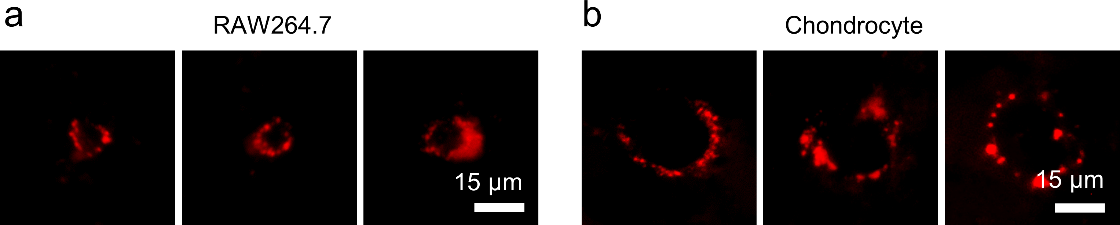


**Figure S2 MNPs-TRPV1 specifically binds to the plasma membrane. (a, b)** Fluorescence images of Cy3-conjugated MNPs-TRPV1 bound to the plasma membrane of RAW264.7 cells **(a)** and chondrocytes **(b)**.


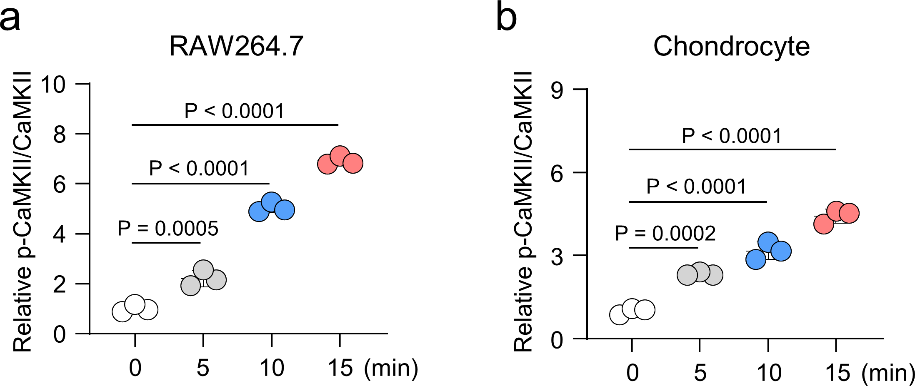


**Figure S3** Quantification of the ratio of protein expression levels of p-CaMKII to CaMKII in RAW264.7 cells **(a)** and chondrocytes **(b)** that induced by MNPs-TRPV1 and AMF for distinct times. One-way ANOVA with Tukey’s post-hoc test. Data are shown as mean ± SD.


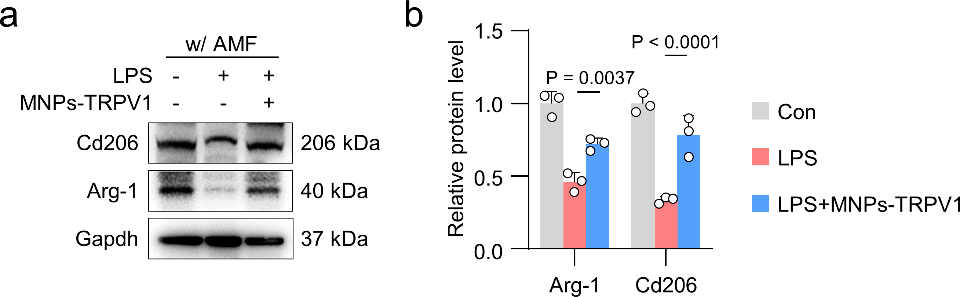


**Figure S4** Western blot measurement **(a)** and quantification **(b)** of proteins resisting macrophagic inflammation in RAW264.7 cells treated as indicated. w/ , with. One-way ANOVA with Tukey’s post-hoc test. Data are shown as mean ± SD.


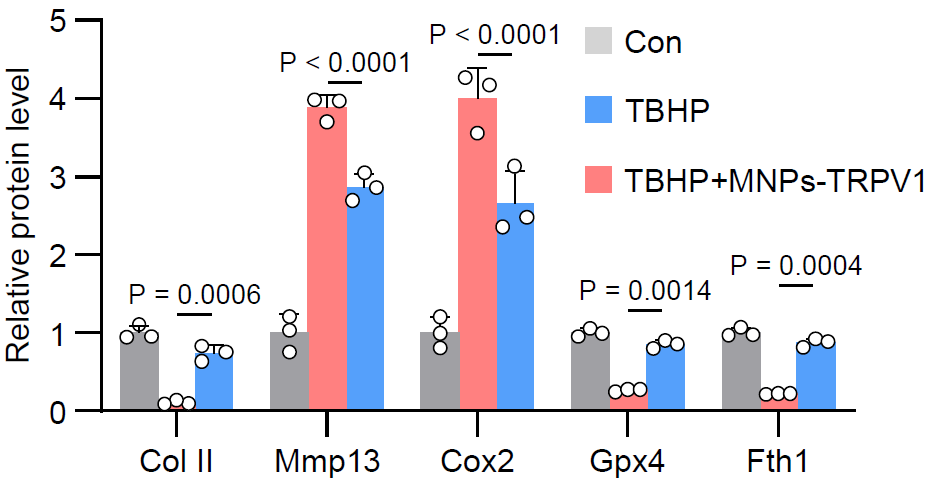


**Figure S5** Quantification of protein expression levels in chondrocytes induced by TBHP with or without MNPs-TRPV1 under AMF treatment. One-way ANOVA with Tukey’s post-hoc test. Data are shown as mean ± SD.


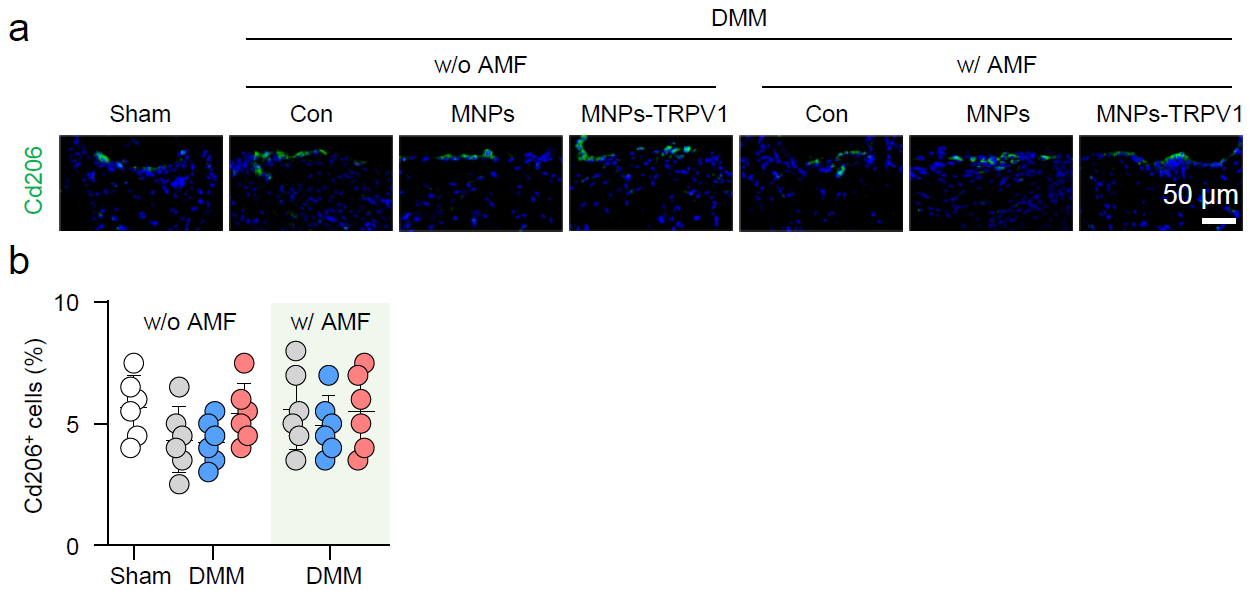


**Figure S6** Representative images (a) and quantification (b) of immunofluorescence staining of anti-macrophagic inflammation marker Cd206 expressed in the synovium of mice treated as indicated. w/o, without; w/ , with. One-way ANOVA with Tukey’s post-hoc test. Data are shown as mean ± SD.


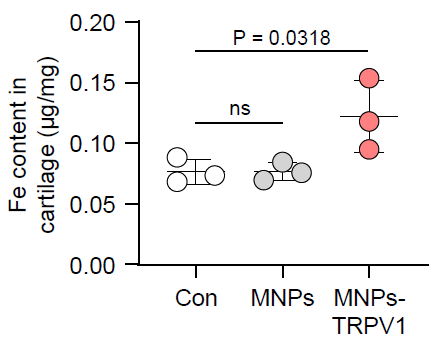


**Figure S7** ICP-MS detection revealed significantly increased iron content in cartilage three weeks after intra-articular MNPs-TRPV1 injection, comparing to MNPs, suggesting at least one week retention of MNPs-TRPV1 in the articular cartilage. One-way ANOVA with Tukey’s post-hoc test. Data are shown as mean ± SD.


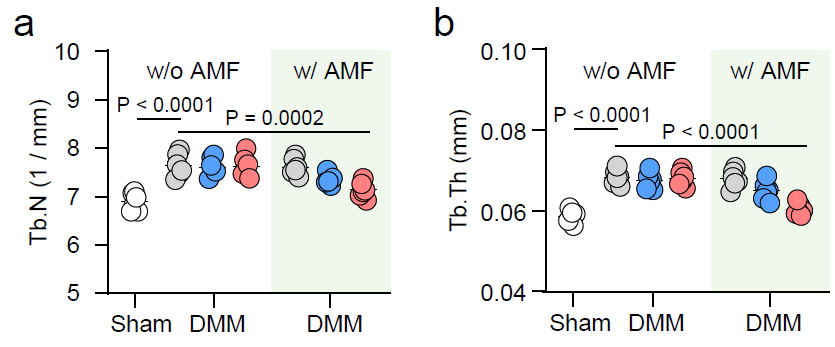


**Figure S8** Quantification of the number (Tb.N) and thickness (Tb.Th) of trabecular of the subchondral bone in mice treated as indicated. w/o, without; w/ , with. One-way ANOVA with Tukey’s post-hoc test. Data are shown as mean.

**Table S1**

Table S1. The primers sequences used in qPCR analysis.

| **Gene** | **Forward primer (5’→3’)** | **Reverse primer (5’→3’)** |
| --- | --- | --- |
| Primers used in RAW264.7 cells to measure macrophagic inflammation | | |
| ***Il-1β*** | TGCCACCTTTTGACAGTGATG | AAGGTCCACGGGAAAGACAC |
| ***Il-6*** | TAGTCCTTCCTACCCCAATTTCC | TTGGTCCTTAGCCACTCCTTC |
| ***Tnf-α*** | CCACGTCGTAGCAAACCACC | GATAGCAAATCGGCTGACGG |
| ***Il-18*** | GAAAGCCGCCTCAAACCTTC | GGTGGATCCATTTCCACTTTGA |
| ***Ptgs2*** | TGAGCAACTATTCCAAACCAGC | GCACGTAGTCTTCGATCACTATC |
| ***Gapdh*** | CCGCATCTTCTTGTGCAGTG | CGATACGGCCAAATCCGTTC |
| Primers used in primary chondrocytes to evaluate ferroptosis | | |
| ***Gpx4*** | AAAGTCCTAGGAAACGCCCG | CTGTTGCAGTACTGGGGAGG |
| ***Fth1*** | TGCCTCCTACGTCTATCTGTC | GTCATCACGGTCTGGTTTCTTT |
| ***Slc7a11*** | CACTGCCATGGTCAGAAAGC | GCATAGGACAGGGCTCCAAA |
| ***Cd44*** | TCGATTTGAATGTAACCTGCCG | CAGTCCGGGAGATACTGTAGC |
| ***Ptgs2*** | TGAGCAACTATTCCAAACCAGC | GCACGTAGTCTTCGATCACTATC |
| ***Ncoa4*** | CCTGGGGCAATCTGAAGGG | CTGAGGAGTCACCAACCAATC |
| ***Cdo1*** | GGGCACGGCAGCAGTATTC | GGCTGACGTTCTCTACTCGG |
| ***Atf3*** | GTCACCAAGTCTGAGGCGG | GTTTCGACACTTGGCAGCAG |
| ***Pgd*** | TGAAGGGTCCTAAGGTGGTCC | CCGCCATAATTGAGGGTCCAG |
| ***Tfrc*** | GTTTCTGCCAGCCCCTTATTAT | GCAAGGAAAGGATATGCAGCA |
| ***Col2a1*** | CCTCAAGGCAAAGTTGGTCCT | CTCCCGTCTCACCGTCTTTT |
| ***Mmp13*** | TCTTTATGGTCCAGGCGATGA | ATCAAGGGATAGGGCTGGGT |
| ***Gapdh*** | CCGCATCTTCTTGTGCAGTG | CGATACGGCCAAATCCGTTC |
